# Incidence and risk factors for adhesive small bowel obstruction after gastric cancer surgery

**DOI:** 10.1007/s00423-026-04032-3

**Published:** 2026-03-28

**Authors:** Yuto Kawate, Hiroshi Kusanagi

**Affiliations:** Department of Gastroenterological Surgery, Kameda Medical Center, 929 Higashi-cho, Kamogawa-shi, Chiba 296-8602 Japan

**Keywords:** Gastrectomy, intestinal obstruction, laparoscopy, risk factors, stomach neoplasms

## Abstract

**Purpose:**

Adhesive small bowel obstruction is a significant complication following gastric cancer surgery; however, its long-term incidence and risk factors remain unclear. This study aimed to clarify the incidence and risk factors for adhesive small bowel obstruction after gastric cancer resection based on long-term follow-up data.

**Methods:**

We conducted a retrospective cohort study of 2,738 patients who underwent radical gastrectomy for gastric cancer at our institution between January 1993 and December 2022. The development of adhesive small bowel obstruction was set as the endpoint. The association with clinical factors was evaluated using the Kaplan-Meier method and Cox proportional hazards regression analysis.

**Results:**

During a median follow-up period of 5.8 years (interquartile range 2.4–11.7 years), 195 patients (7.1%) developed adhesive small bowel obstruction. Among them, 69 cases (35.4%) were reported within the first year after surgery, whereas 31 cases (15.9%) developed after 10 years. Multivariate analysis identified ileus during hospitalization, open surgical approach (hazards ratio [HR] 2.16), male sex, blood loss ≥ 224 mL, and history of abdominal surgery as independent risk factors (HRs: 2.86, 2.16, 1.93, 1.89, and 1.46, respectively).

**Conclusion:**

Adhesive small bowel obstruction after gastric cancer surgery can develop beyond the conventional 5-year follow-up period, and the risk persists even after 10 years. Minimizing surgical invasiveness through laparoscopic approaches and reduced blood loss may lower the risk of adhesive small bowel obstruction.

## Introduction

Adhesive small bowel obstruction (ASBO) is a representative long-term complication following gastrointestinal surgery that not only diminishes patients’ quality of life but also imposes significant medical and economic burdens through rehospitalization and repeated surgeries [1, 2]. Previous studies reported that the incidence of ASBO after gastric cancer surgery ranges from approximately 11.7%–38.5% [3]; however, large-scale long-term observational studies on its timing and risk factors are limited. Pan et al. [4] conducted a single institution retrospective cohort study of the risk factors and incidence of ASBO; however, their study had a relatively short follow-up period. Thus, more detailed studies with longer follow-up periods are necessary.

The standard follow-up period after gastric cancer surgery is typically 5 years. However, the long-term course of late complications such as ASBO has not been sufficiently examined. Therefore, this study aimed to elucidate the incidence of ASBO and its associated clinical factors using a large cohort with an observation period spanning 30 years.

## Materials and methods

### Study population

**Fig. 1:**
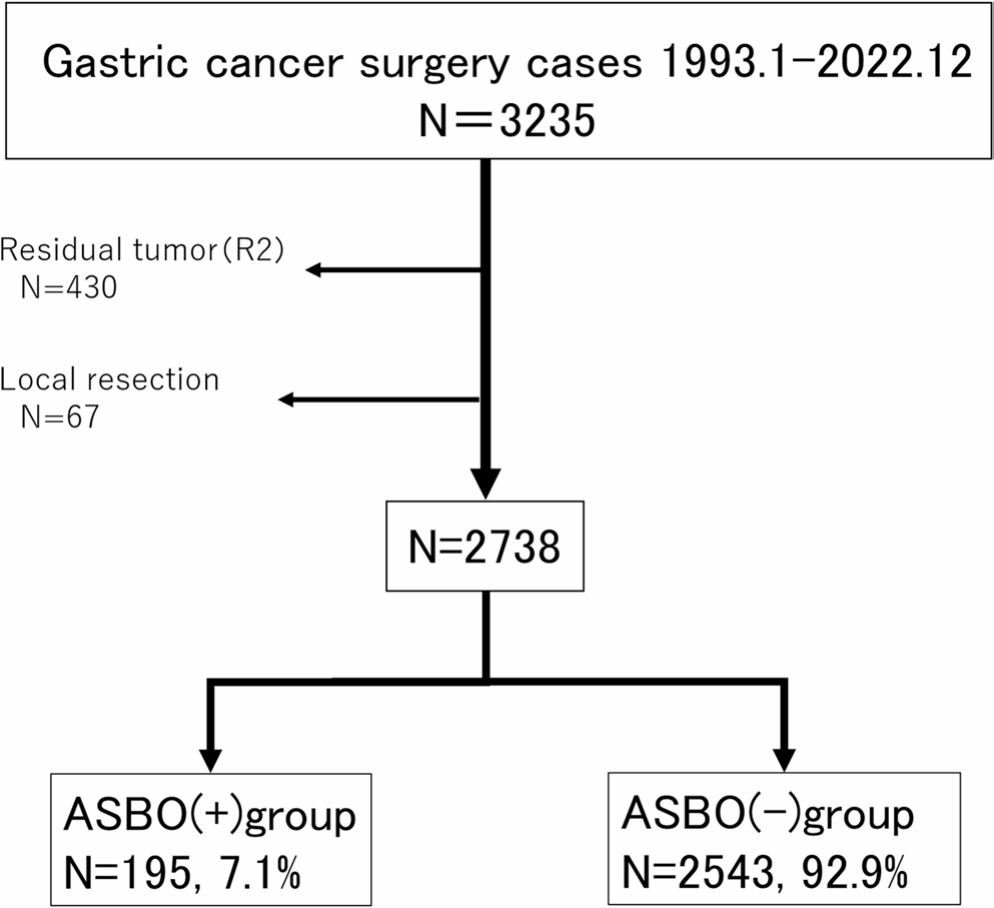
Flowchart of patient selection

This study included the data of 3,235 patients who underwent gastrectomy for gastric cancer at Kameda Medical Center between January 1993 and December 2022. After excluding local resection cases (67 patients) and non-curative resection cases (430 patients), the data of 2,738 patients were included for analysis (Fig. 1).

From 3,235 gastric cancer surgery cases performed between January 1993 and December 2022, the data of 2,738 patients were included in the final analysis after excluding 430 non-curative resection cases and 67 local resection cases. Among the patients analyzed, 195 (7.1%) patients developed adhesive small bowel obstruction (ASBO).

### Ethical approval

The protocol for this research project has been approved by a suitably constituted Ethics Committee of the institution (Ethics Review Committee of Kameda Medical Center, Approval No. 23–110). The study was performed in accordance with the ethical standards as laid down in the 1964 Declaration of Helsinki and its later amendments or comparable ethical standards. Exemption from informed consent was granted through an opt-out method.

### Clinical data collection

The following information was extracted from the medical records: age, sex, American Society of Anesthesiologists physical status classification system, pathological TNM stage (according to the 15th edition of the Japanese Classification of Gastric Carcinoma [5]), surgical procedure, surgical approach, extent of lymph node dissection, presence of postoperative intra-abdominal infection, occurrence of ileus during hospitalization, perioperative blood transfusion, history of abdominal surgery, Charlson Comorbidity Index, operative time, blood loss, body mass index, and reconstruction method.

Postoperative intra-abdominal infection was defined as encapsulated abscesses in the abdominal cavity detected by postoperative computed tomography scans and requiring antibiotic treatment, including anastomotic leakage and pancreatic fistula. Ileus during hospitalization was defined as any intestinal passage disturbance, including postoperative paralytic ileus. ASBO was defined as a post-discharge presentation with symptoms such as abdominal pain and vomiting, small bowel dilatation on abdominal computed tomography, and requiring hospitalization. Cases of obstruction due to anastomotic stenosis or cancer recurrence were excluded.

### Statistical analysis

The interval between surgery and the first occurrence of ASBO was examined. The association between clinical factors and ASBO development was analyzed using univariate analysis with the Kaplan-Meier method and multivariate analysis with Cox proportional hazards regression. Continuous variables were dichotomized using cutoff values determined by the maximum area under the receiver operating characteristic curve. *P*-values < 0.05 were considered statistically significant. All statistical analyses were performed with EZR (Jichi Medical University, Tochigi, Japan), which is a graphical user interface for R (The R Foundation for Statistical Computing, Vienna, Austria). More precisely, this software is a modified version of R commander designed to add statistical functions frequently used in biostatistics [6].

## Results

### Patient characteristics

Of the 2,738 patients, 1,925 (70.3%) were male and 813 (29.7%) were female, with a median age of 69 years (interquartile range [IQR] 60–76 years). The stage distributions were as follows: Stage I, 1,589 cases (58.0%); Stage II, 523 cases (19.1%); Stage III, 513 cases (18.7%); and Stage IV, 111 cases (4.1%). The surgical procedures performed were distal gastrectomy in 1,931 patients (70.5%), total gastrectomy in 771 (28.2%), and proximal gastrectomy in 36 (1.3%). The surgical approach was open in 2,195 patients (80.2%) and laparoscopic in 543 (19.8%). Additional clinical characteristics of the patients are shown in Table 1.

**Table 1:** Clinical characteristics of patients with and without small bowel obstruction

Characteristic	Total (*n* = 2738)	SBO+ (*n* = 195)	SBO- (*n* = 2543)	*P* value
Follow-up period, median days (IQR)	2069 (834–5108)	4416 (2082–7030)	1994 (801–4983)	< 0.001
Age, median (IQR)	69 (60–76)	66 (58–73)	69 (61–76)	< 0.001
Gender, n (%)				0.001
Male	1925 (70.3)	159 (81.5)	1766 (69.4)	
Female	813 (29.7)	36 (18.5)	777 (30.6)	
ASA-PS, n (%)				< 0.001
1	345 (12.6)	40 (20.5)	305 (12.0)	
2	1892 (69.1)	135 (69.2)	1757 (69.1)	
3	481 (17.6)	19 (9.7)	462 (18.2)	
4	6 (0.2)	0 (0)	6 (0.2)	
Cancer Stage, n (%)				0.034
I	1589 (58.0)	131 (67.2)	1458 (57.3)	
II	523 (19.1)	34 (17.4)	489 (19.2)	
III	513 (18.7)	26 (13.3)	487 (19.2)	
IV	111 (4.1)	4 (2.1)	107 (4.2)	
Gastrectomy type, n (%)				0.011
Distal gastrectomy (DG)	1931 (70.5)	120 (61.5)	1811 (71.2)	
Total gastrectomy (TG)	771 (28.2)	73 (37.4)	698 (27.4)	
Proximal gastrectomy (PG)	36 (1.3)	2 (1.0)	34 (1.3)	
Surgical approach, n (%)				< 0.001
Laparoscopic	543 (19.8)	14 (7.2)	529 (20.8)	
Open	2195 (80.2)	181 (92.8)	2014 (79.2)	
Lymph node dissection, n (%)				0.053
< D2	955 (34.9)	55 (28.2)	900 (35.5)	
≥ D2	1775 (64.8)	139 (71.3)	1636 (64.5)	
IAIC, n (%)				0.061
No	2585 (94.4)	178 (91.3)	2407 (94.7)	
Yes	151 (5.5)	17 (8.7)	134 (5.3)	
Ileus during hospitalization, n (%)				0.003
No	2681 (97.9)	185 (94.9)	2496 (98.2)	
Yes	55 (2.0)	10 (5.1)	45 (1.8)	
Another neoplasm resection, n (%)				0.502
No	2663 (97.3)	188 (96.4)	2475 (97.5)	
Yes	71 (2.6)	7 (3.6)	64 (2.5)	
Blood transfusion, n (%)				0.014
No	2278 (83.2)	173 (88.7)	2105 (82.8)	
Yes	433 (15.8)	18 (9.2)	415 (16.3)	
Previous abdominal operation, n (%)				0.035
No	1651 (60.3)	103 (53.6)	1548 (61.6)	
Yes	1053 (38.5)	89 (46.4)	964 (38.4)	
CCI grade, n (%)				0.004
Low	1629 (59.5)	137 (71.7)	1492 (59.8)	
Medium	850 (31.0)	48 (25.1)	802 (32.2)	
High	186 (6.8)	5 (2.6)	181 (7.3)	
Very high	32 (1.2)	1 (0.5)	31 (1.2)	
Operation time, median min (IQR)	226 (190–273)	240 (208–280)	225 (190–273)	0.001
Blood loss, median mL (IQR)	265 (130–480)	380 (250–646)	260 (120–470)	< 0.001
BMI, median kg/m² (IQR)	22.5 (20.4–24.7)	23.1 (21.2–25.0)	22.4 (20.3–24.7)	0.013
Reconstruction, n (%)				0.030
Billroth I (B1)	1719 (62.8)	104 (53.3)	1615 (63.5)	
Billroth II (B2)	117 (4.3)	8 (4.1)	109 (4.3)	
Roux-en-Y (RY)	853 (31.2)	79 (40.5)	774 (30.4)	
Others	49 (1.8)	4 (2.1)	45 (1.8)	

*SBO* small bowel obstruction, *IQR* interquartile range, *ASA-PS* american society of anesthesiologists physical status, *IAIC* intra-abdominal infectious complication, *CCI* comprehensive complication index, *BMI* body mass index, Reconstruction Others = jejunal interposition, double tract reconstruction, and other modified techniques

*P* values < 0.05 were considered statistically significant

### Interval to first ASBO occurrence

**Fig. 2:**
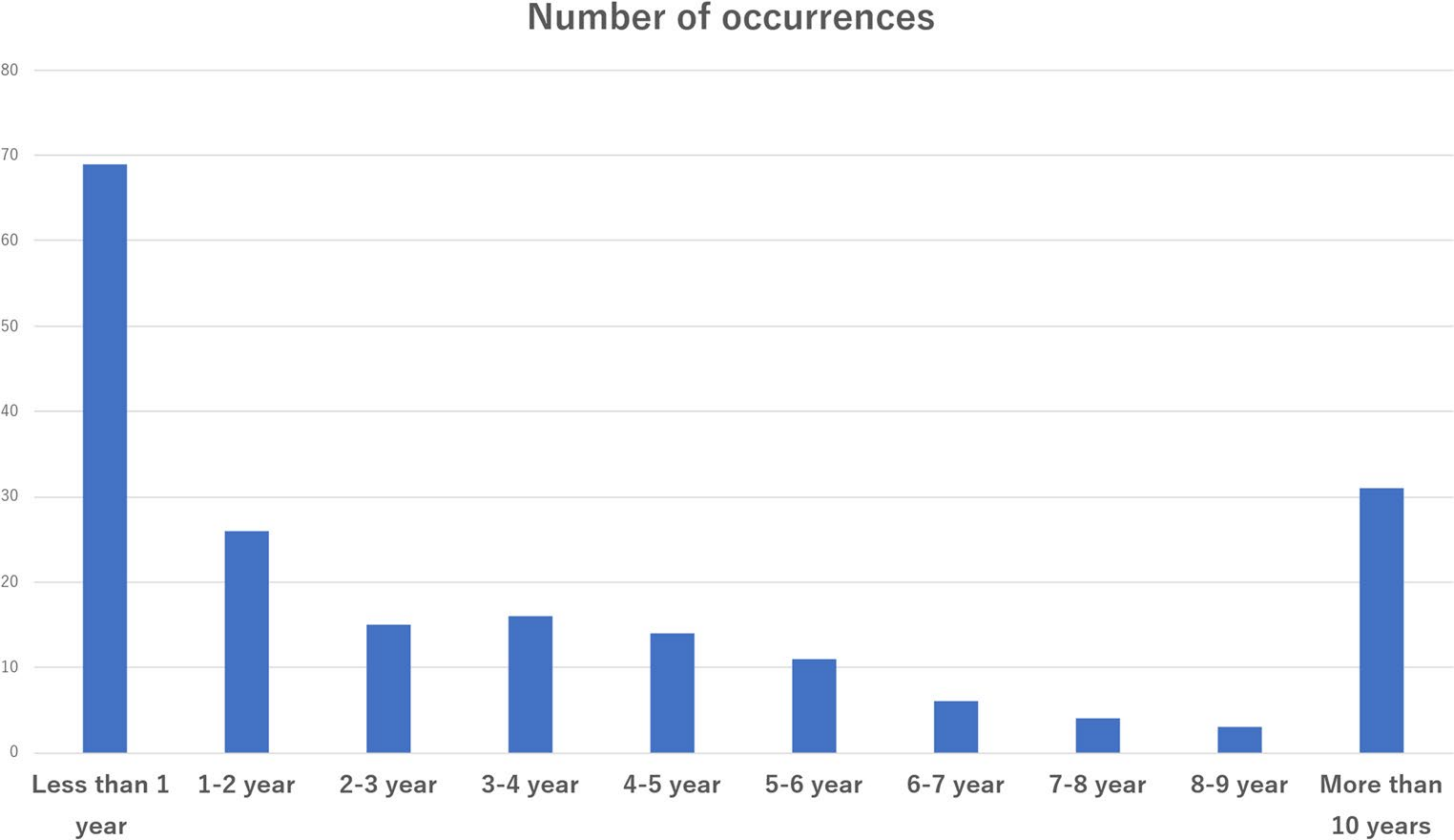
Chronological distribution of adhesive small bowel obstruction (ASBO) after gastric cancer surgery. The highest occurrence was within the first year after surgery (69 cases, 35.4%); however, ASBO continued to develop beyond the conventional 5-year follow-up period, with 31 cases (15.9%) occurring more than 10 years postoperatively

During a median follow-up period of 5.8 years (IQR 2.4–11.7 years), 195 patients (7.1%) developed ASBO. Among them, the highest proportion occurred within the first year after surgery (69 patients, 35.4%). Subsequently, 26 patients (13.3%) developed ASBO in 1–2 years, 15 (7.7%) in 2–3 years, 16 (8.2%) in 3–4 years, and 14 (7.2%) in 4–5 years, indicating a gradual decrease in the number of cases over time. However, cases continued to occur beyond 5 years post-surgery, with 31 cases (15.9%) developing after 10 years (Fig. 2).

### Long-term ASBO development beyond 10 years

A total of 847 patients had a follow-up period exceeding 10 years. Among these patients, 120 (14.2%) developed ASBO at some point during the entire follow-up period, and 31 (3.7%) developed ASBO for the first time more than 10 years after surgery. Of the 31 patients with late-onset ASBO, 5 (16%) had undergone additional abdominal surgery after their initial gastrectomy.

### Risk factors for ASBO development

Univariate analysis identified male sex (*p* < 0.001), type of gastrectomy (*p* < 0.001), open surgical approach (*p* < 0.001), extent of lymph node dissection (*p* = 0.042), postoperative intra-abdominal infection (*p* = 0.013), ileus during hospitalization (*p* < 0.001), history of abdominal surgery (*p* = 0.033), operative time ≥ 208 min (*p* = 0.005), blood loss ≥ 224 mL (*p* < 0.001), and reconstruction method (*p* < 0.001) as significant risk factors for ASBO development (Table 2).

**Table 2:** Log-rank analysis of clinical factors associated with small bowel obstruction

Risk Factor	*N*	5-year Incidence (%)	*P* value
Sex			< 0.001
Male	1925	7.3	
Female	813	3.9	
Age			0.300
< 75 years	1925	0.6	
≥ 75 years	813	0.6	
ASA-PS			0.154
1	345	8.8	
2	1892	6.1	
3	481	4.4	
4	6	0.0	
Cancer Stage			0.980
I	1589	6.1	
II	523	7.0	
III	513	5.8	
IV	111	5.5	
Gastrectomy type			< 0.001
Distal gastrectomy (DG)	1931	5.3	
Total gastrectomy (TG)	771	9.0	
Proximal gastrectomy (PG)	36	4.3	
Surgical approach			< 0.001
Laparoscopic	543	2.5	
Open	2195	7.3	
Lymph node dissection			0.042
< D2	955	5.2	
≥ D2	1775	6.8	
IAIC			0.013
No	2585	5.8	
Yes	151	14.9	
Ileus during hospitalization			< 0.001
No	2681	6.0	
Yes	55	21.8	
Blood transfusion			0.268
No	2287	6.3	
Yes	433	4.9	
Previous abdominal operation			0.033
No	1651	5.5	
Yes	1053	7.5	
CCI grade			0.251
Low	1629	6.5	
Medium	850	6.3	
High	186	3.5	
Very high	32	3.6	
Operation time			0.005
< 208 min	1010	4.5	
≥ 208 min	1719	7.3	
Blood loss			< 0.001
< 224 mL	1159	2.9	
≥ 224 mL	1571	8.2	
BMI			0.056
< 18.5 kg/m²	263	3.0	
18.5–24.9 kg/m²	1825	6.1	
≥ 25 kg/m²	629	7.5	
Reconstruction			< 0.001
Billroth I (B1)	1719	5.2	
Billroth II (B2)	117	5.4	
Roux-en-Y (RY)	853	8.5	
Others	49	9.3	

*SBO *small bowel obstruction, *ASA-PS* american society of anesthesiologists physical status, *IAIC* Intra-Abdominal infectious complication, *CCI *charlson comorbidity index, *BMI* body mass index, reconstruction others = include jejunal interposition, double tract reconstruction, and other modified techniques.

*P* values < 0.05 were considered statistically significant

Multivariate analysis identified five independent risk factors for ASBO: ileus during hospitalization (hazard ratio [HR] 2.86, 95% confidence interval [CI] 1.38–5.90, *p* = 0.005), open surgical approach (HR 2.16, 95% CI 1.13–4.15, *p* = 0.020), male sex (HR 1.93, 95% CI 1.32–2.83, *p* < 0.001), blood loss ≥ 224 mL (HR 1.89, 95% CI 1.24–2.87, *p* = 0.003), and history of abdominal surgery (HR 1.46, 95% CI 1.09–1.97, *p* = 0.012) (Table 3).

**Table 3:** Multivariate analysis of risk factors for small bowel obstruction using Cox proportional hazards regression model

Risk Factor	Category	Hazard Ratio	95% CI	*P* value
Age				
< 75 years	Reference	1.000	-	-
≥ 75 years		1.103	0.740–1.646	0.630
Sex				
Female	Reference	1.000	-	-
Male		1.929	1.316–2.828	< 0.001
ASA-PS				
1	Reference	1.000	-	-
2		0.811	0.554–1.187	0.281
3		0.685	0.350–1.338	0.268
4		0.000	0.000-Inf	0.992
CCI grade				
Low	Reference	1.000	-	-
Medium		0.801	0.558–1.150	0.228
High		0.361	0.128–1.018	0.054
Very high		0.923	0.121–7.034	0.938
BMI				
< 18.5 kg/m²	Reference	1.000	-	-
18.5–24.9 kg/m²		1.930	0.887–4.197	0.097
≥ 25 kg/m²		1.636	0.723–3.706	0.238
Previous abdominal operation				
No	Reference	1.000	-	-
Yes		1.462	1.087–1.967	0.012
Cancer Stage				
I	Reference	1.000	-	-
II		0.774	0.517–1.160	0.214
III		0.785	0.496–1.242	0.301
IV		0.537	0.167–1.728	0.297
Gastrectomy type				
Distal gastrectomy (DG)	Reference	1.000	-	-
Total gastrectomy (TG)		1.165	0.647–2.105	0.613
Proximal gastrectomy (PG)		0.857	0.119–6.150	0.879
Reconstruction				
Billroth I (B1)	Reference	1.000	-	-
Billroth II (B2)		1.068	0.490–2.330	0.868
Roux-en-Y (RY)		1.247	0.698–2.228	0.456
Others		1.214	0.289–5.160	0.793
Surgical approach				
Laparoscopic	Reference	1.000	-	-
Open		2.162	1.126–4.150	0.020
Lymph node dissection				
< D2	Reference	1.000	-	-
≥ D2		1.166	0.813–1.672	0.402
IAIC				
No	Reference	1.000	-	-
Yes		1.495	0.863–2.587	0.151
Ileus during hospitalization				
No	Reference	1.000	-	-
Yes		2.858	1.384–5.898	0.005
Blood loss				
< 224 mL	Reference	1.000	-	-
≥ 224 mL		1.887	1.241–2.869	0.003
Operation time				
< 208 min	Reference	1.000	-	-
≥ 208 min		1.105	0.766–1.593	0.593

*SBO* small bowel obstruction, *ASA-PS* american society of anesthesiologists physical status, *CCI* charlson comorbidity index, *BMI* body mass index; Reconstruction Others = include jejunal interposition, double tract reconstruction, and other modified techniques; IAIC = Intra-Abdominal infectious complication

Hazard ratios were calculated using Cox proportional hazards regression model. *P* values < 0.05 were considered statistically significant

## Discussion

This study revealed that ASBO development after gastric cancer surgery persists well beyond the conventional follow-up period of 5 years, with 15.9% of cases occurring more than 10 years postoperatively. The independent risk factors identified by multivariate analysis suggest that surgical invasiveness, represented by blood loss and surgical approach, plays a crucial role in ASBO development. Furthermore, the strong correlation between ileus during hospitalization identified.

Our findings can be compared with the study by Pan et al. [4], which reported on ASBO incidence and risk factors following gastrectomy for gastric cancer at a high-volume institution in China. Both studies identified open surgical approach and history of abdominal surgery as common independent risk factors for ASBO, thereby reinforcing the robustness of these specific findings across different patient populations and healthcare settings.

However, there were notable differences in the other identified risk factors between the two studies. Our study identified male sex, intraoperative blood loss (≥ 224 mL), and ileus during hospitalization as independent risk factors, whereas Pan et al. identified reconstruction method, extent of lymphadenectomy, combined organ resection, and postoperative intraabdominal complication as independent risk factors. These differences may reflect variations in patient populations, surgical practices, and analytical approaches between the two institutions.

Several key differences distinguish our study from those of Pan et al. First, our study encompasses a longer maximum follow-up period of 30 years. This extended observation allowed us to specifically characterize long-term ASBO development: among 847 patients with a follow-up period exceeding 10 years, 120 (14.2%) developed ASBO during the entire follow-up period, and 31 (3.7%) developed ASBO for the first time more than 10 years after surgery. This finding provides concrete evidence that ASBO risk persists well beyond the conventional 5-year surveillance period, a point that has not been previously documented with such specificity.

Second, our study identified ileus during hospitalization as the strongest independent risk factor for ASBO development (HR 2.86, 95% CI 1.38–5.90), which was not reported in the study by Pan et al. This novel finding suggests that early postoperative intestinal dysfunction may serve as a predictive marker for future ASBO risk, highlighting the importance of careful management of postoperative ileus and potentially identifying a subgroup of patients who may benefit from closer long-term surveillance.

Third, our data originate from a Japanese healthcare setting, complementing the Chinese cohort reported by Pan et al. The consistency of certain risk factors across these two East Asian populations strengthens the external validity of these specific findings.

Adhesion formation is regulated by complex biochemical interactions involving inflammation, angiogenesis, and tissue repair processes [7]. Regarding the relationship between surgical invasiveness and ASBO development, the identification of open surgery as a risk factor suggests that the minimal invasiveness of laparoscopic surgery contributes to ASBO prevention. This is consistent with previous reports indicating that laparoscopic surgery reduces postoperative adhesions [8, 9]. Additionally, increased blood loss was identified as an independent risk factor. In the supplementary analysis of “JCOG1001”, a randomized controlled trial investigating the association between the antiadhesion membrane and small bowel obstruction after open gastrectomy, a large amount of blood loss was independently associated with ASBO development [10]. Clinicians and researchers widely acknowledge that the fibrinolytic system plays a central role in the pathophysiology of adhesion formation. Experimental evidence suggests that inflammatory mediators, such as transforming growth factor-β and interleukins, reduce the fibrinolytic capacity of the peritoneum, thereby increasing adhesion formation [11–14]. Blood loss may serve as an indicator of the degree of surgical invasiveness and tissue damage, potentially contributing to adhesion formation through enhanced inflammatory responses.

Regarding the association between ileus during hospitalization and ASBO development, early intestinal dysfunction may trigger an inflammatory response in the intestinal wall, promoting adhesion formation. This association underscores the importance of appropriate management of postoperative early intestinal peristaltic disturbances.

Notably, male sex was identified as a significant independent patient-related risk factor. Although the detailed mechanism underlying this correlation has not yet been fully elucidated, previous studies have shown male sex as a risk factor for paralytic ileus after colorectal cancer surgery [15].

Other reports have shown that visceral obesity serves as a risk factor for the development of postoperative ileus after colorectal cancer surgery [16]. Possible mechanisms include the anatomical characteristic of relatively greater intra-abdominal fat accumulation in males, which may promote postoperative adhesion formation. Further research on the molecular basis of sex-related adhesion formation mechanisms is warranted.

The strength of this study lies in its large single-institution cohort with a long-term observation period spanning 30 years, which has elucidated the ASBO development pattern over a longer period than that in previous studies. Our institution serves an extensive medical catchment area; with no alternative medical institutions in the region, residents rely on our hospital for healthcare needs. This geographical situation has facilitated the collection of comprehensive patient data.

Limitations of this study include its single-center retrospective design and the inability to eliminate the influence of changes in treatment policies over time. The imbalance between open surgery (80.2%) and laparoscopic surgery (19.8%) reflects the historical evolution of surgical techniques during the 30-year study period. Laparoscopic gastrectomy was introduced at our institution in 1998, and its adoption has gradually increased over time. Although multivariate analysis adjusted for surgical approach, this imbalance may limit the generalizability of our findings regarding the protective effect of laparoscopic surgery. Future studies with more balanced cohorts or propensity score matching analyses would be valuable to further validate these findings. Furthermore, this study did not systematically account for additional abdominal surgeries that may have occurred during the follow-up period. However, in a supplementary analysis of the 31 patients who developed ASBO more than 10 years after gastrectomy, only 5 patients (16%) had undergone additional abdominal surgery after their initial gastric cancer surgery. This suggests that the majority of late-onset ASBO cases occurred without intervening abdominal procedures. Nevertheless, the potential contribution of subsequent surgeries to adhesion formation in the broader cohort cannot be entirely excluded.

## Conclusion

This study demonstrated that the risk of ASBO after gastric cancer surgery persists well beyond the conventional 5-year follow-up period. As new cases continue to occur even after 10 years postoperatively, clinicians should be mindful of potential ASBO risk during long-term follow-up after gastric cancer surgery. Minimizing surgical invasiveness, including the choice of laparoscopic approach and strategies for blood loss reduction, is important to reduce the risk of ASBO. Careful postoperative management and longer-term follow-up are recommended, particularly for patients with multiple risk factors.

## Data Availability

The data that support the findings of this study are available from the corresponding author upon reasonable request.

## Declarations

### Permission to reproduce material from other sources

N/A.

### Clinical trial registration

N/A.
